# Systematically Characterizing Chemical Profile and Potential Mechanisms of Qingre Lidan Decoction Acting on Cholelithiasis by Integrating UHPLC-QTOF-MS and Network Target Analysis

**DOI:** 10.1155/2019/2675287

**Published:** 2019-01-03

**Authors:** Peng Huang, Hongwen Ke, Yang Qiu, Mingchen Cai, Jialin Qu, Aijing Leng

**Affiliations:** ^1^Clinical Laboratory of Integrative Medicine, The First Affiliated Hospital of Dalian Medical University, No. 222, Zhongshan Road, Dalian 116011, China; ^2^Institute of Integrative Medicine, Dalian Medical University, No. 9, South Road of Lvshun, Dalian 116044, China; ^3^Department of Traditional Chinese Medicine, The First Affiliated Hospital of Dalian Medical University, No. 222, Zhongshan Road, Dalian 116011, China; ^4^Central Laboratory, The First Affiliated Hospital of Dalian Medical University, No. 222, Zhongshan Road, Dalian 116011, China

## Abstract

Qingre Lidan Decoction (QRLDD), a classic precompounded prescription, is widely used as an effective treatment for cholelithiasis clinically. However, its chemical profile and mechanism have not been characterized and elucidated. In the present study, a rapid, sensitive, and reliable ultraperformance liquid chromatography coupled with quadrupole time-of-flight mass spectrometry method was established for comprehensively identifying the major constituents in QRLDD. Furthermore, a network pharmacology strategy based on the chemical profile was applied to clarify the synergetic mechanism. A total of 72 compounds containing flavonoids, terpenes, phenolic acid, anthraquinones, phenethylalchohol glycosides, and other miscellaneous compounds were identified, respectively. 410 disease genes, 432 compound targets, and 71 related pathways based on cholelithiasis-related and compound-related targets databases as well as related pathways predicted by the Kyoto Encyclopedia of Genes and Genomes database were achieved. Among these pathways and genes, pathway in cancer and MAPK signaling pathway may play an important role in the development of cholelithiasis. EGFR may be a crucial target in the conversion of gallstones to gallbladder carcinoma. Regulation of PRKCB/RAF1/MAP2K1/MAPK1 is associated with cell proliferation and differentiation. Thus, the fingerprint coupled with network pharmacology analysis could contribute to simplifying the complex system and providing directions for further research of QRLDD.

## 1. Introduction

Traditional Chinese Medicine possess a history of thousands of years, which has been widely used in clinical practice in China and played an increasingly important role to health maintenance and disease treatment. Traditional Chinese Formula (TCF) is the main form of clinical application of Traditional Chinese Medicine. Due to its satisfactory clinical efficacy, TCF has been regarded as an alternative and promising medicine strategy for treating complex diseases all over the world [1]. Qingre Lidan Decoction (QRLDD) is a classic precompounded prescription, which contains 6 herbs, namely, Lysimachiae Herba (jin-qian-cao in Chinese), Scutellariae Radix (huang-qin in Chinese), Aurantii Fructus (zhi-qiao in Chinese), Aucklandiae Radix (mu-xiang in Chinese), Gardeniae Fructus (zhi-zi in Chinese), and Rhei Radix et Rhizoma (da-huang in Chinese). It has been extensively applied in clinical treatment of cholecystitis and gallstones for many years with the satisfactory therapeutic effects in several hospitals [2, 3]. The main mechanism of its efficacy has been reported to relax sphincter of Oddi, promote bile excretion, and prevent stagnation [4]. However, the current research on QRLDD has two drawbacks: firstly, a clear understanding of the relationship between ingredient and formula has not been elucidated; secondly, in aspect of pharmaceutical effect, current reports usually focus on the level of single inflammatory mediator or protein, which is hardly to reflect the characteristic of multicomponents and multitargets of Chinese medicine formula [5]. These are obstacles for the development and the therapeutic efficacy of QRLDD.

In recent years, the rapid development of network pharmacology has provided a novel method for revealing the molecular mechanisms associated with the therapeutic efficacy of multicomponent in TCF [6]. It has facilitated understanding the interactions of ingredient, target, and disease systematically based on systems biology, polypharmacology, and molecular network analysis, rather than an individual target [7]. Thus, the application of network pharmacology provides a powerful and promising method for analyzing TCF.

**Figure 1:**
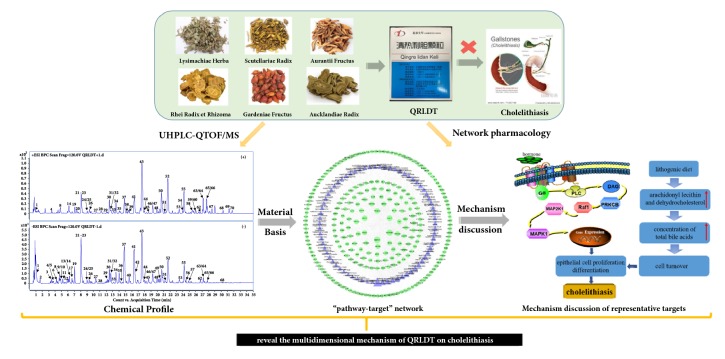
Schematic diagram of present study.

The schematic diagram of present study was shown in Figure 1; an ultraperformance liquid chromatography coupled with quadrupole time-of-flight mass spectrometry (UHPLC-QTOF-MS) method was established to analyze the major chemical constituents of QRLDD in this present study. Potential targets and related pathways were correspondingly explored by using network pharmacology method based on the identified components, and the mechanism of QRLDD in the treatment of cholelithiasis was elucidated systematically.

## 2. Materials and Methods

### 2.1. Chemicals, Reagents, and Materials

UHPLC–MS grade acetonitrile and methanol were purchased from Merck Company Inc. (Darmstadt, Germany) and MS grade formic acid was supplied by Fisher Scientific Company Inc. (Fairlawn, NJ). Ultrapure water (18.2 MΩ) was prepared with a Milli-Q water purification system (Millipore, Milford, MA, USA). All other reagents were of analytical grade and purchased from Tianjin Concord Technology Co. Ltd. (Tianjin, China)

The reference compounds gallic acid (**2**), protocatechuic acid (**3**), 4-hydroxybenzoic acid (**10**), (+) catechin (**13**), chlorogenic acid (**15**), caffeic acid (**17**), syringing (**20**), geniposide (**21**), (-)-epicatechin (**22**), rutin (**29**), kaempferol (**36**), hesperidin (**40**), neohesperidin (**41**), baicalin (**43**), quercetin (**47**), baicalein (**55**), aloe-emodin(**60**), rhein (**61**), wogonin (**64**), emodin (**68**), dehydrocostuslactone (**70**), chrysophanol (**71**), and physcion (**72**) were purchased from the National Institutes for Food and Drug Control (Beijing, China). The purity of each reference standard was determined to be over 98% by UHPLC analysis. All the 6 herbs of QRLDD, including Lysimachiae Herba, Scutellariae Radix, Aurantii Fructus, Aucklandiae Radix, Gardeniae Fructus, and Rhei Radix et Rhizoma, were purchased from the first affiliated hospital of Dalian Medical University (Dalian, Liaoning Province, China), and authenticated by Professor Aijing Leng (Department of Chinese medicine, The First Affiliated Hospital of Dalian Medical University). Voucher specimens were deposited at the authors' laboratory.

### 2.2. Preparation of Samples and Standard Solution

The QRLDD samples were prepared by the decocting method. A blended mixture of Lysimachiae Herba (30 g), Scutellariae Radix (15 g), Aucklandiae Radix (15 g), Aurantii Fructus (15 g), and Gardeniae Fructus (15 g) was soaked in 10-fold mass of water (900 mL) for 1 h and boiled for 1 h and then filtered with six-layer absorbent gauze. An 8-fold mass of water (800 mL) was subsequently added to residues and boiled for 30 min. Then Rhei Radix et Rhizoma (10 g) was added into the extract and boiled for additional 30 min. After being filtered with six-layer absorbent gauze, the two filtrates were combined and concentrated under vacuum to 100 mL (equal to 1 g crude herb/mL), and finally the concentrate was transformed into the freeze-dried powder.

A 1.0 g of the freeze-dried powder was accurately weighted and extracted with 50 mL of methanol/water (1:1, v/v) for 30 min under ultrasound. The extract solution was centrifuged at 13000 rpm for 10 min at 4°C, and the supernatant was filtered through a 0.22 *μ*m filter. 1.0 *μ*L of filtrate was injected to UHPLC-QTOF-MS for analysis.

### 2.3. Chromatography and MS Conditions

Chromatographic separation was performed on an Agilent 1290 Infinity LC system (Agilent, USA) using an Agilent Zorbax Eclipse Plus C18 column (100 × 2.1 mm i.d., 3.5 *μ*m). The oven temperature was maintained at 40°C. Water containing 0.1% formic acid (solvent system A) and acetonitrile (solvent system B) served as the mobile phase. The gradient elution program was 0–5 min, 3%–10% B; 5–13 min, 10%–18% B; 13–20 min, 18%–25% B; 20–28 min, 25%–35% B; 28 to 33 min, 35% to 99% B; 33–35 min, 99%–3% B; 35–40 min, 3% B.

Mass detection was performed using an Agilent 6530b Accurate-Mass Quadrupole Time-of-Flight (Q-TOF) mass spectrometer (Agilent Corp., USA) equipped with a Dual AJS ESI source operating in both positive and negative mode with the following operating parameters: drying gas (N_2_) flow rate, 10.0 L/min; drying gas (N_2_) temperature, 350°C; nebulizer, 35 psig; sheath gas (N_2_) temperature, 400°C; fragmentor voltage, 120 V; skimmer voltage, 65 V; Octopole RF, 750 V. The capillary voltage was set at 4 kV or –3.5 kV under positive or negative mode, respectively. The nozzle voltage was set at +500 V or –1000 V, respectively; four collision energies at 10 V, 20 V, 30 V, and 40 V were applied to acquire sufficient product ions. MS spectra were recorded over the m/z range of 50–1100. All data was processed by MassHunter workstation software version B.06.00 (Agilent Technologies, Germany).

### 2.4. Target Network Pharmacology Analysis

#### 2.4.1. Therapeutic Targets of Cholelithiasis

Cholelithiasis associated targets were obtained from six existing resources: (1) TTD database (http://bidd.nus.edu.sg/BIDD-Databases/TTD/TTD.asp), which could provide a comprehensive information platform about the clinical trial drugs, targets and pathways [8]; (2) OMIM database (http://omim.org/), which catalogues all known diseases with a genetic component and provides references for further research and tools for genomic analysis of a catalogued gene [9]; (3) PharmGKB database (https://www.pharmgkb.org/), which provides a various array of PGx information, from annotations of the primary literature to guidelines for adjusting drug treatment based on genetic information [10]; (4) DrugBank database (http://www.drugbank.ca/, version 4.3), which includes >4100 drug entries, >14 000 protein or drug target sequences that relevant to these drug entries [11]; (5) GAD database (https://geneticassociationdb.nih.gov/), which provides a platform analysis for complex common human genetic disease systematically [12]. (6) DisGeNET database (http://www.disgenet.org/web/DisGeNET/menu), which offers available collections of genes and variants related to human diseases [13].

We searched these databases with keywords “cholecystitis”, “acute cholecystitis”, “chronic cholecystitis”, “gallstones”, “cholangitis”, “jaundice”, “obstructive jaundice” and got 410 genes totally after removing duplicates. The detailed information is provided in Supplementary Table S1.

#### 2.4.2. Compound Target for QRLDD

After identifying the compounds contained in QRLDD by UHPLC-QTOF-MS/MS, the InChI Key, Canonical SMILES, and CAS number of compounds were obtained from NCBI PubChem database (https://www.ncbi.nlm.nih.gov/pubmed/). And ingredient-related targets were accordingly collected from the Traditional Chinese Medicine Systems Pharmacology Database and Analysis Platform (TCMSP) (http://lsp.nwu.edu.cn/tcmsp.php) and Swiss Target Prediction (http://www.swisstargetprediction.ch/) with their names and/or CAS number as key words. Then, their official symbol was obtained after input of the targets with the species limited to “Homo sapiens” via UniProtKB (http://www.uniprot.org/) [14]. Finally, genes information of ingredients was achieved. The details are supplied in Supplementary Table S2.

#### 2.4.3. The Protein–Protein Interactions (PPIs) Network Analysis

The protein–protein interactions (PPIs) network was constructed and analyzed by STRING database. In order to further identify the primary therapeutic targets to guarantee the accuracy of results, only those PPIs with high confidence score (>0.95) were selected for network construction and analysis [15].

#### 2.4.4. Network Construction and Analysis

All the networks can be performed by utilizing the network visualization software Cytoscape 3.2.1 [16], which supplies a method for data integration, analysis, and visualization for complicated network analysis. Three networks were constructed as follows: (1) protein-protein interactions (PPIs) of cholelithiasis targets; (2) herb-compound-compound targets network of QRLDD; (3) pathways-targets network analysis. In this network plot, a “node” signifies an herb, ingredient, or gene; an “edge” represents interaction among different targets. The “degree” of a node was in agreement with the number of its connected edges [17].

#### 2.4.5. Enrichment Analysis

To clarify the pathways that are relate to putative QRLDD targets, Kyoto Encyclopedia of Genes and Genomes (KEGG) pathway aenrichments bsed on Database for Annotation, Visualization and Integrated Discovery (DAVID, https://david.ncifcrf.gov/home.jsp, ver. 6.8) were applied [18].

## 3. Results and Discussion

### 3.1. Chemical Profile of QRLDD by UHPLC-QTOF-MS

In the present study, a specific UHPLC-ESI-QTOF MS^n^ protocol was performed to rapidly identify the compounds of QRLDD based on the optimized LC and MS conditions systemically.

**Figure 2:**
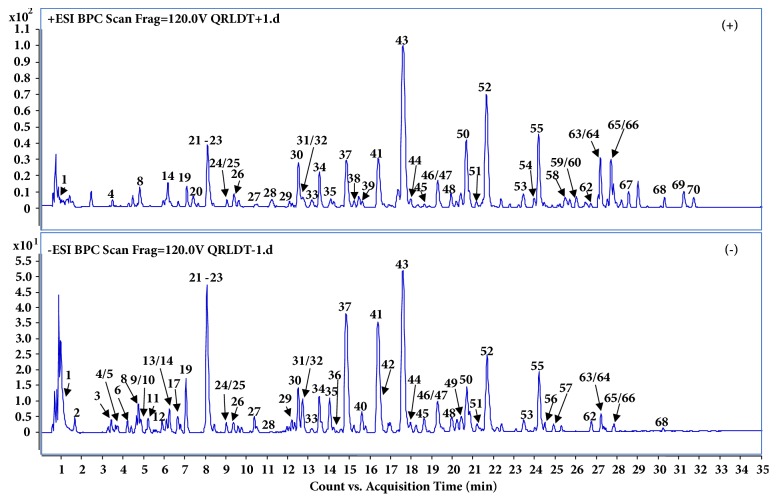
Representative base peak chromatogram (BPC) of QRLDD in the positive and negative ions mode, respectively. See Table 1 for the peak numbers, and see Section 2.3* Chromatography and MS conditions* for UHPLC-QTOF-MS conditions.

As a result, a total of 72 compounds, including 33 flavonoids, 17 terpene, 9 phenolic acid, 5 anthraquinones, 3 phenethylalchohol glycosides, and 5 miscellaneous compounds were identified or tentatively characterized (Figure 2, Table 1). Among them, 23 constituents (compounds** 2**-**3**,** 10**,** 13**,** 15**,** 17**,** 20**-**22**,** 29**,** 36**,** 40**-**41**,** 43**,** 47**,** 55**,** 60**-**61**,** 64**,** 68,** and** 70**-**72**) were unambiguously identified as gallic acid, protocatechuic acid, 4-hydroxybenzoic acid, (+)-catechin, chlorogenic acid, caffeic acid, syringin, geniposide, (-)-epicatechin, rutin, kaempferol, hesperidin, neohesperidin, baicalin, quercetin, baicalein, aloe-emodin, rhein, wogonin, emodin, dehydrocostuslactone, chrysophanol, and physcion by direct comparison of their retention time and MS Spectra with reference compounds, respectively. For the compounds without chemical standards, the molecular formula was established by high-accurate quasi-molecular ion such as [M−H]^−^, [2M−H]^−^, [M+Cl]^−^, [M+HCOO]^−^, [M+H]^+^ and [M+Na]^+^ within a mass error of 10.0 ppm, fractional isotope abundance, and their fragmentation patterns with related literatures. Information regarding the 72 constituents, such as t_R_ (min), identification, formula, negative ion (m/z), positive ion (m/z), and source, is offered in Table 1, and the exact identification of each group of components is outlined in Table 1 and Figure 2.

#### 3.1.1. Identification of Flavones

A total of 33 flavones and their glycosides were screened from Scutellariae Radix, Gardeniae Fructus, Aurantii Fructus, and Lysimachiae Herba of QRLDD, with 9 of them unambiguously elucidated and the other tentatively identified. With respect to the glycosides, their MS spectra afforded the aglycone product due to the cleavage at the glycosidic linkage, with 146 Da, 162 Da, and 176 Da as the characteristic neutral loss of rhamnosyl, glucosyl, and glucuronic acid residues, respectively. MS^2^ spectra with high energy showed characteristic ^1,3^ A^−^ and ^1,3^ B^−^ ions origin from a retro-Diels-Alder (RDA) cleavage of C ring as well as losses of CH_3_ (15 Da), CO (28 Da), H_2_O (18 Da), CO_2_ (44 Da), and/or combination of the fragments above-mentioned.

**Figure 3:**
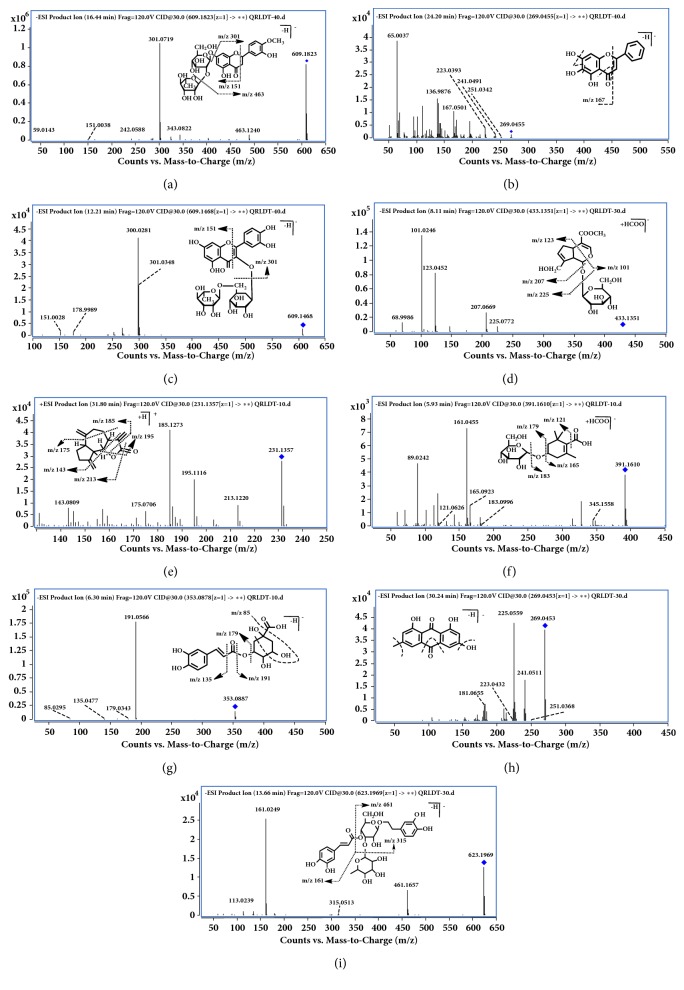
QTOF-ESI-MS/MS spectra and proposed fragmentation pathways of neohesperidin (a), baicalein (b), rutin (c), geniposide (d), jasminoside B (f), chlorogenic acid (g), emodin (h), and acteoside (i) in negative ion mode and dehydrocostuslactone (e) in positive ion mode.

*(1) Dihydroflavones.* A total of seven dihydroflavones were identified from QRLD samples, with peaks** 35**,** 40, **and** 41** definitely elucidated and the others tentatively assigned. Peaks** 40** and** 41** were accurately identified as hesperidin and neohesperidin by compared with their respective references. Corresponding to the previous paper [19], high-accurate quasi-molecular ions of peak** 41** were obtained in negative ion mode at m/z 609.1823, which was identified as hesperidin. The quasi-second-order precursor ions at m/z 301.0719 and 463.1240 were generated from m/z 609.1823 ([M-H]^−^), suggesting continuous losses of glucosyl (162 Da) and rhamnosyl (146 Da). The most dominate ions at m/z 151 and m/z 149 were yielded from m/z 301.0719 owning to RDA reaction by breaking two C-C bonds of C-ring (Figure 3(a)). Similarly, Peak** 35** exhibited the [M-H]^−^ ion at m/z 579.1709 (C_27_H_32_O_14_, retention time 14.09 min) as well as the ions at m/z 151 and m/z 119 yielded from m/z 271.0621[M-H-glc-rha]^−^ through RDA reaction. The latter was 30 Da (-CH_2_O) lower than that of Peak** 41**. Therefore, it was identified as narirutin, a methoxy-substituted derivative at C-6 position, according to the above information and literature [20]. Correspondingly, peaks** 31**,** 32**,** 37, **and** 53** were tentatively assigned as carthamidin, neoeriocitrin, naringin, and hesperetin based on in-house library for QRLDD and further fragmentation patterns mentioned above.

*(2) Flavones and Their Glycosides*. Twenty-five flavones and their glycosides were unambiguously or tentatively identified. Peak** 55**, a representative major constituent in QRLDD, was taken as an example. It displayed quasi-molecular ion [M–H]^−^ at m/z 269.0455 and was unequivocally identified as baicalein in comparison with an authentic standard. In the MS/MS spectrum, characteristic fragment ions m/z 251, 241, and 223 were formed by successive losses of H_2_O (18 Da) and CO (28 Da), while the most dominant ions at m/z 167.0501 were yielded through RDA reaction (Figure 3(b)).

Similarly, peak** 43 (**definitely identified as baicalin) displayed a quasi-molecular ion [M–H]^−^ at m/z 445.0773 and aglycone ion (m/z 269) that resulted from the loss of a glucuronic acid (176 Da) by easy cleavage of glycosidic bond. With similar fragmentation patterns as baicalein, fragment ions at m/z 251, 241, 223, and 167 were also detected. Thus, the fragmentation features of* O*-linked glycosyls and fragment ions of aglycones were applied in the characterization of the remaining flavones glycosides

In addition, cyclization reaction was also observed in part of flavones and their glycosides.

Peak** 29** was selected as the example for the stepwise elucidation of this appearance. It was identified as rutin by comparing with authentic standard, which exhibited quasi-molecular ion [M-H]^−^ at m/z 609.1466. Its MS^2^ spectra gave the ions at m/z 463.0896 and m/z 301.0346, indicating the successive loss of rhamnose and rutinose, while, except for similar skeleton with baicalein (Peak** 55**), m/z 178 and m/z 151 generated by cyclization reaction after RDA reaction in the C ring were also observed in the MS/MS spectrum (Figure 3(c)). Analogically, the other compounds were tentatively assigned following this fragmentation pathway and related literatures.

#### 3.1.2. Identification of Terpenes

Seventeen terpenoids, including nine iridoids and their glycosides, three sesquiterpenoids, three diterpenes, and two monoterpenes, were screened from QRLDD. Among them, peaks** 21** and** 70** were unambiguously identified as geniposide and dehydrocostuslactone by comparison with reference standards.

*(1) Iridoids and Their Glycosides*. Peak** 21** exhibited [M+HCOO]^−^ ion at m/z 433.1351 (C_17_H_24_O_10_, retention time 8.11 min) in negative ion mode. It produced characterized MS^2^ fragment ions at m/z 225, m/z 207, m/z 123, and m/z 101 owing to the glycosidic linkage, further dehydration at C_1_ and C_9_ positions, and RDA reaction between C_1_-O_2_ and C_4_-C_5_, respectively (Figure 3(d)). Similarly, peak** 9 **with a [M+HCOO]^−^ ion at m/z 449.1300 (C_17_H_24_O_11_, retention time 3.58 min) was 16 Da (+O) higher than quasi-molecular ion of peak** 21**. It also produced a desugarization ion at m/z 225.0772. Its predominant fragment ions at m/z 139 and m/z 101 were obtained owing to the RDA reaction. The former was 16 Da (+O) higher than that of Peak** 21**. Thus, this compound was tentatively assigned as scandoside methyl ester according to publications [21]. Analogously, the remaining compounds were tentatively identified by comparison of their retention behavior and MS/MS spectrum with the literature date [21, 22].

*(2) Sesquiterpenoids*. Two distinct peaks** 69** and** 70 **with [M+H]^+^ ions at m/z 233.1535 and 231.1357 were observed in positive ion mode, respectively. Their most probable molecular formulas were inferred to be C_15_H_20_O_2_ and C_15_H_18_O_2_ according to exact molecular weight. Compound** 70** was identified as dehydrocostuslactone by comparison with its standard. Its tandem mass spectra and possible fragmentation pathway was illustrated in Figure 3(e). It showed the protonated ion at m/z 231.1357. The fragment ions at m/z 213, 185, 157, 195, and 175 were the characteristic behavior owing to successive neutral losses of H_2_O, CH_2_O_2_, C_3_H_6_O_2_, H_2_O_4_, and C_4_H_8,_ respectively [23]. Compound** 69** was accordingly identified as costus lactone in a similar way. In addition, Peak** 59** from Scutellariae radix was observed in negative ion mode and identified as dikamaliartanes A on the basis of MS data and related literature [22].

*(3) Diterpenes and Monoterpenoid Glycoside*. Three diterpenes were detected in QRLDD in negative ion mode. Peak** 48 **gave an [M-H]^−^ ion at m/z 813.3186 and showed fragment ions at m/z 651, 489, and 327 by simultaneous losses of glucosyl groups (162 Da), which was deduced to crocin-2 based on the exact molecular formulae matching, fragmentation, and literature date [22]. Peak** 44** and** 56** exhibited the same [M-H]^−^ ion at m/z 975.3715 (C_44_H_64_O_24_, retention times 18.03 and 24.49 min), which was 162 Da (+C_6_H_10_O_5_) higher than that of peak** 48**. They also showed the same fragments ions with Peak** 48**. By matching the constructed compound library, they were deduced to crocin-1 and crocin-4, a pair of cis-trans isomer originated from Gardeniae Fructus. In addition, as the polarity of cis-diterpenes was larger than that of trans-diterpenes, peaks** 44** and** 56** were identified a**s **crocin-1 and crocin-4, respectively [22, 24].

Two monoterpenoids from Gardeniae Fructus were tentatively identified and their cleavage pathway is similar to that of iridoid glycosides with slightly differences. The losses of glycosides (162 Da), CO_2_ (44 Da), and H_2_O (18 Da) were the characteristic fragmentations in their MS^2^ spectra [22, 25]. Peak** 12** was selected as the example for the stepwise elucidation of the molecular structure. It yielded the ions at m/z 183.0996 and m/z 165.0923, which corresponded to successive losses of a glycoside and H_2_O, respectively. The former further produced a fragment ion at m/z 121.0626 [M-H-glc-CO_2_]^−^. Consequently, Peak** 12** was reasonably deduced to be jasminoside B according to aforementioned fragmental information and reference data (Figure 3(f)) [22]. Peak** 39** was tentatively assigned as jasminoside S/H/I following this fragmentation pathway; however, it needed to be confirmed by the reference standards.

#### 3.1.3. Identification of Phenolic Acids

Nine phenolic acids, originated from Scutellariae Radix, Lysimachiae Herba, and Gardeniae Fructus, were detected as minority of components in QRLDD. The negative ion mode was much more suitable for their analysis. Peaks** 2**,** 3**,** 10**,** 15,** and** 17** were unambiguously identified as gallic acid, protocatechuic acid, 4-hydroxybenzoic acid, chlorogenic acid, and caffeic acid by comparison with authentic references. Peaks** 16** and** 24** were tentatively identified as darendoside A and p-coumaric acid on the basis of the exact molecular formulae matching, fragmentation, and the literature date [22, 26]. Take chlorogenic acid (Peak** 15**) for example. Its MS chromatograms exhibited a quasi-molecular ion at m/z 353.0890 [M−H]^−^ as well as two diagnostic fragment ions at m/z 191.0563 (loss of a caffeoyl group, 162 Da) and 179.0343 (loss of a quinic acid, 174 Da). Another fragment ion at m/z 135.0477 was formed by the neutral losses of CO_2_ (44 Da) via the break of ester bond in caffeic acid. In addition, m/z 85.0295 formed via the breaks of C_3_−C_4_ and C_5_−C_6_ as well as successive neutral loss of CO_2_ was also observed (Figure 3(g)) [22]. Additionally, peaks** 7** and** 18** exhibited the [M-H]^−^ ions at m/z 353.0882 and 353.0883 with molecular formula speculated as C_16_H_18_O_9_, the fragment ions at m/z 191.0227 and 179.0357 were the same as chlorogenic acid (**15**), suggesting that they should be isomers of chlorogenic acid. Tao et al. reported that three isomeric neochlorogenic acid, chlorogenic acid, and cryptochlorogenic acid were contained in Gardeniae Fructus [27]. Moreover, the retention time for chlorogenic acid was later and earlier than that of neochlorogenic acid and cryptochlorogenic acid in a similar UHPLC system, respectively [28]. Therefore, peaks** 7** and** 18** were tentatively identified as neochlorogenic acid and cryptochlorogenic acid, respectively.

#### 3.1.4. Identification of Anthraquinones

Five anthraquinones were unambiguously identified by comparison with authentic references, which were more suitable for the analysis in negative ion mode. Successive or simultaneous neutral losses of H_2_O, CO, O, and CH_3_ were the characteristic behavior of this type of compounds. Peak** 68** (t_R_ = 30.24 min) was selected as an example, which displayed the [M-H]^−^ ion at m/z 269.0453. The yield ion at m/z 241.0511 was formed by direct loss of the CO, followed by the loss of O, and gave the ion at m/z 225. 0559. The fragment ion of m/z 181, 251, and 223 was corresponded to the losses of CO_2_, H_2_O, and CO, respectively (Figure 3(h)). Similarly, aloe-emodin, rhein, chrysophanol, and physcion were elucidated [24].

#### 3.1.5. Identification of Phenethylalchohol Glycosides

Three phenethylalchohol glycosides were tentatively identified due to the absence of reference standards, which were from Scutellariae Radix and Lysimachiae Herba. Caffeic acid, hydroxytyrosol, and glycosyls were the basic groups of this type of compounds. Peak** 34 **was selected as the example for the stepwise elucidation. Peak** 34** with the quasi-molecular ion m/z 623.1969 and product ions at m/z 461.1657, m/z 315.1010, and m/z 161.0249 were detected in the MS/MS spectrum. The product ions were generated from m/z 623.1981 by loss of a caffeoyl group (162 Da), m/z 461.1675 by loss of rhamnosyl residue (146 Da), and m/z 179.0353 by elimination of H_2_O (18 Da), respectively (Figure 3(i)). It was identified as acteoside in consistent with the fragment information of literature [29]. Analogously, the remaining peaks** 46** and** 50 **were tentatively identified as isomers cistanoside D and cistanoside C following above fragmentation pathway and polarity feature [29].

#### 3.1.6. Other Types of Miscellaneous Compounds

Other compounds (peaks** 1**,** 11**,** 20**,** 58,** and** 62**) were tentatively assigned as galloyl glucose, procyanidin B_2_, syringin, meranzin, and limonin, respectively, on the basis of the exact molecular formulae matching, fragmentation information as well as the literature data [30–32] but still need to be further confirmed by reference standard.

### 3.2. Target Identification and Network Analysis

#### 3.2.1. Cholelithiasis-Related Targets Network Analysis

**Figure 4:**
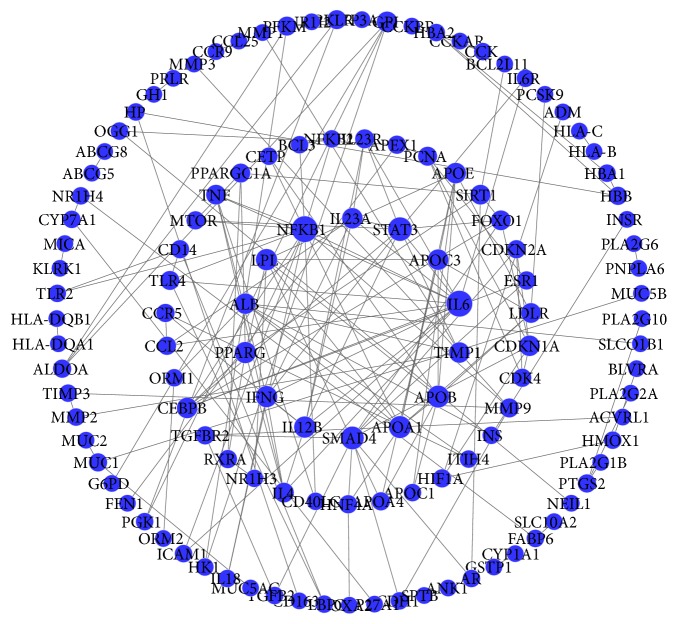
*Cholelithiasis-related targets *PPI network (confidence score >0.95).

The relationship among 410 disease genes from PPI was extracted by STRING. And a gene-gene interaction network was accordingly constructed. 122 nodes and 173 edges were involved in this network (Figure 4). Among them, the nodes located at the central part (IL6, NFKB1 and STAT3) connected by more edges have higher degree, such as 13 in IL6, 13 in NFKB1, and 10 in STAT3. It implies that these genes may be the important targets in the formation and development of cholelithiasis.

#### 3.2.2. Herb-Compound-Compound Targets Network Analysis

**Figure 5:**
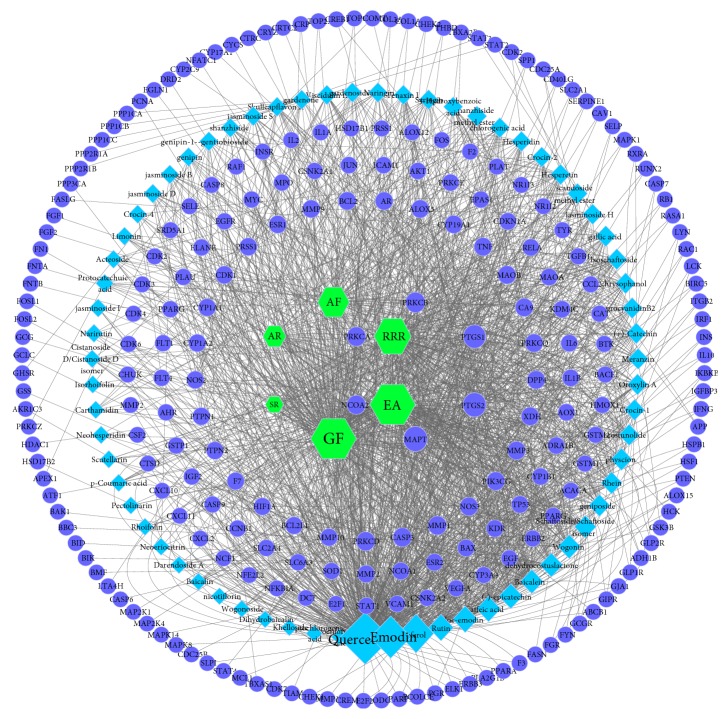
Herb-compound-compound target network of QRLDD (blue circle represents compound targets, cyan diamond represents for compound, and green hexagon represents herb; node size represents the degree).

**Figure 6:**
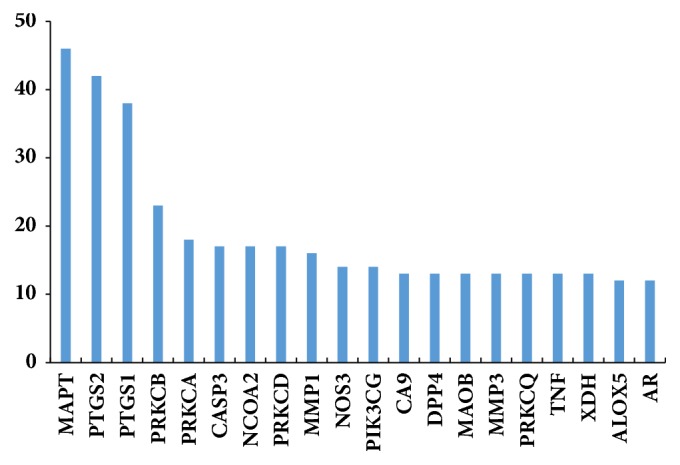
Degree of top 20 compound targets.

The relationship among 432 compound targets from PPI were constructed and analyzed by STRING. Compound targets of PPI with high confidence score (>0.95) were screened. And herb-compound-compound targets network constructed by cytoscape was shown in Figure 5, which comprises 313 nodes (6 herb nodes, 67 compound nodes, and 240 compound target nodes) and 1937 edges. From this network, we can conclude that Gardeniae Fructus, lysimachiae Herba, and Scutellariae Radix may be the main herbs in treating disease due to their higher degree. According to the frequency statistics of 77 Chinese medicine cases on gallstones, Lysimachiae Herba, Scutellariae Radix, Aurantii Fructus, and Aucklandiae Radix were used for 55, 47, 21, and 12 times, respectively [33]. We can also find that many compounds acting on the same target and multiple targets contacted by the same compound. For example, MAPT is the targets of aloe-emodin, geniposide, gallic acid, and other chemical components. Quercetin simultaneously acts on IL10, MAPK1, HSF1, among many other targets. However, some can be regulated by only one compound, such as CA9, which is simply controlled by Khelloside. The degree of top 20 targets was listed in Figure 6.

The result indicted that compounds from QRLDD may act on these targets systematically and play an important pharmacological role in treating cholelithiasis, which is in line with herbal formulae's feature of multicompound and multi-target. The potential mechanism can be elucidated by this network.

#### 3.2.3. Pathway of QRLDD-Disease Network

**Figure 7:**
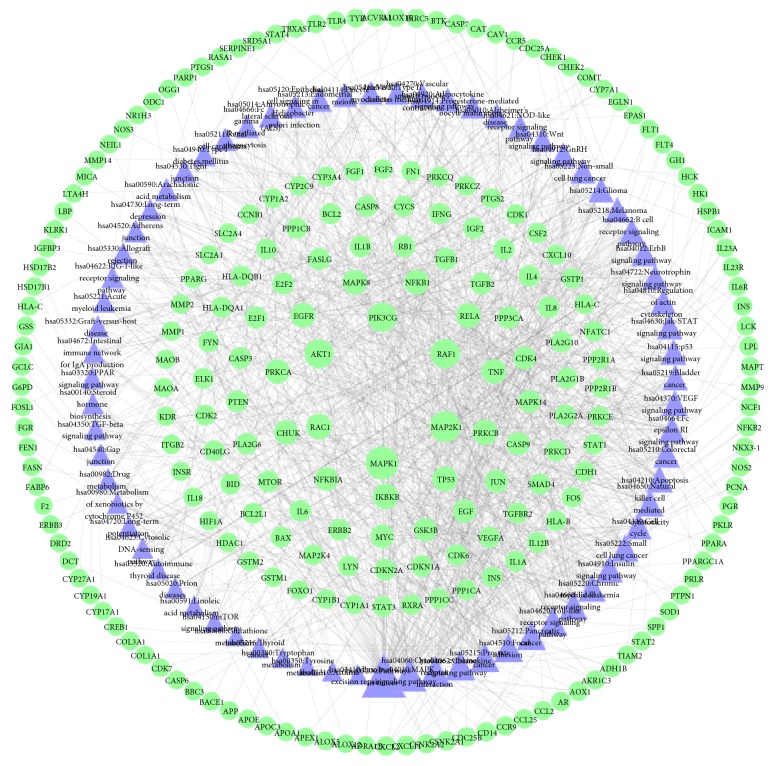
Illustration of relations among chemical constituent targets and involved pathways of QRLDD (green circle represents compound target, blue triangle represents pathway, green hexagon represents herb, and purple hexagon represents pathway. Node size represents the degree).

In order to better understand the mechanism of QRLDD on cholelithiasis, 71 related pathways (P<0.5) were obtained by inputting all targets into DAVID; the details are described in Supplementary Table S3. As shown in Figure 7, Pathway in cancer (hsa05200) is ranked first, which has 72 genes involved; among them, PTGS2, TP53, and IL6 have a higher degree. The result is consistent with clinical data, which confirmed the close relationship between gallstones and gallbladder cancer [34]. Within the screened genes, EGFR was selected for example, whose expression is associated with proliferation, differentiation, lymphatic metastasis, and other processes of gallbladder carcinoma [35]. Therefore, the analysis of pathways in cancer will help to understand the pathogenesis of gallbladder carcinoma caused by gallstones and provide basis for the future study.

**Figure 8:**
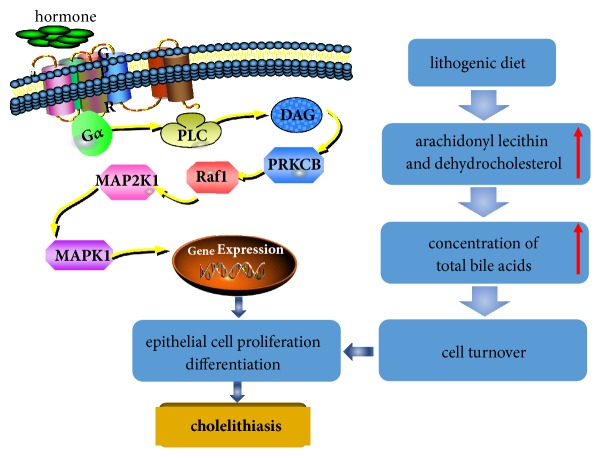
Mechanism of key targets screened by network in the formation of cholelithiasis.

In addition, there were 41, 35, and 31 pathways associated with MAPK1, MAP2K1, and RAF1, among which, MAPK signaling pathway (hsa04010), chemokine signaling pathway (hsa04062), and Focal adhesion (hsa04510) were the most closely related ones. KEGG data visualization made it obvious that the PRKCB downstream target proteins RAF1, MAP2K1 and MAPK1 are key connection points between the MAPK signaling pathway, chemokine signaling pathway and focal adhesion. According to previous study, lithogenic diet is closely related to the development of cholelithiasis, which tends to alter the components in bile with increasing substances such as arachidonyl lecithin and dehydrocholesterol. As an adaptive response to the environment, cell turnover will be emerged [36]. In the development of gallstone formation, mitotic index is shown to increase rapidly at prelithiasic phase [37]. In addition, the condition of gallbladder and bile duct abnormalities has been shown to accelerate the cell turnover and increase cellular proliferating activity [38]. In our present research, regulation of PRKCB/RAF1/MAP2K1/MAPK1 can affect cell proliferation and differentiation (Figure 8). These changes may provide a new perspective for the treatment of cholelithiasis.

Above findings provide a direct connection between metabolic syndrome and cholesterol gallstone. Whether their expression is involved in the curative effect of QRLDD acting on cholelithiasis will be validated in the subsequent research.

## 4. Conclusions

Chinese medicine plays an important role in preventing and treating cholelithiasis. In our study, the chemical profile of Qingre Lidan Decoction was mapped for the first time by UHPLC-QTOF-MS, and 72 ingredients origin from six herbs were attributed. The “multicomponent-multitarget-multipath” mechanism of QRLDD was further explored based on network pharmacology platform in view of the identified ingredient. Our study found that multiple ingredients in QRLDD can exert a combined effect for the same target. Several important targets (EGFR and MAPK1) and pathways (pathways in cancer and MAPK signaling pathway) were predicted to be an important role in the mechanism of QRLDD. The present study not only provide experimental and theoretical basis for the further development and application of QRLDD, but also make beneficial exploration in investigating the molecular synergy of Traditional Chinese Formula.

## Figures and Tables

**Table 1:** Characterization of the chemical constituents in QRLDD by UHPLC–QTOF–MS.

Peak No	t_R_ (min)	Identification	Formula			Negative ion					Positive ion			Source ^*a*^
				Quasi-molecular ion	Observed mass (Da)	Calculated mass (Da)	ppm	Fragment ions	Quasi-molecular ion	Observed mass (Da)	Calculated mass (Da)	ppm	Fragment ions	
1	1.54	Galloyl glucose	C_13_H_16_O_10_	[M-H]^−^	331.0678	331.0671	2.11	287[M-H-CO_2_]	[M+Na]^+^	355.0644	355.0636	2.25	311[M+Na-CO_2_]^+^	RRR
								169[M-H-C_6_H_10_O_5_]					338[M+Na-OH]^+^	
								125[M-H-C_6_H_10_O_5_-CO_2_]					193[M+Na-C_6_H_10_O_5_]^+^	
2 ^*b*^	1.69	Gallic acid	C_7_H_6_O_5_	[M-H]^−^	169.0147	169.0142	2.96	151[M-H-H_2_O]	[M+H]^+^	171.0289	171.0288	0.58	153[M+H-H_2_O]^+^	EA
								108[M-H-CHO_2_]					127[M+H-CO_2_]^+^	
								125[M-H-CO_2_]						
3^* b*^	3.32	Protocatechuic acid	C_7_H_6_O_4_	[M-H]^−^	153.0200	153.0193	4.57	109[M-H-CO_2_]	—	—	—	—		SR/EA
4	3.58	Shanzhiside methyl ester	C_17_H_26_O_11_	[M-H]^−^	405.1400	405.1402	-0.49	361[M-H-CO_2_]	[M+Na]^+^	429.1387	429.1367	4.66		GF
								317[M-H-2CO_2_]						
								225[M-H-gla-H_2_O]						
5	3.68	Shanzhiside	C_16_H_24_O_11_	[M-H]^−^	391.1243	391.1246	-0.77	229[M-H-C_6_H_10_O_5_]	—	—	—	—		GF
								185[M-H-C_6_H_10_O_5_-CO_2_]						
								167[M-H-C_6_H_10_O_5_-H_2_O-CO_2_]						
6	4.26	Gardenoside	C_17_H_24_O_11_	[M+HCOO]^−^	449.1308	449.1301	1.56	403.289[M-H-C_2_H_10_O_5_]	[M+Na]^+^	427.1176	427.1211	-8.19		GF
				[M-H]^−^	403.1265	403.1246	4.71							
7	4.44	Neochlorogenic acid	C_17_H_24_O_11_	[M-H]^−^	353.0882	353.0878	1.13	191[M-H-C_9_H_6_O_3_]	—	—	—	—		GF
8	4.65	Jasminoside D	C_16_H_26_O_8_	[M-H]^−^	345.1568	345.1555	3.77		[M+H]^+^	347.1683	347.1700	-4.90	311[M+H-2H_2_O]^+^	GF
9	4.72	Scandoside methyl ester	C_17_H_24_O_11_	[M+HCOO]^−^	449.1300	449.1301	-0.22	241[M-H-C_6_H_10_O_5_]	[M+Na]^+^	427.1186	427.1211	-5.85		GF
				[M-H]^−^	403.1271	403.1246	6.20							
10 ^*b*^	4.80	4-Hydroxybenzoic acid	C_7_H_6_O_3_	[M-H]^−^	137.0246	137.0244	1.46	93[M-H-CO_2_]	[M+H]^+^	139.0383	139.0390	-5.03		SR/EA
11	5.40	Procyanidin B2	C_30_H_26_O_12_	[M-H]^−^	577.1351	577.1351	0.00	559[M-H-H_2_O]	[M+H]^+^	579.1469	579.1497	-4.83		RRR
								535[M-H-C_2_H_2_O]						
12	5.93	Jasminoside B	C_16_H_26_O_8_	[M+HCOO]^−^	391.1618	391.1610	2.05	183[M-H-C_6_H_10_O_5_]	—	—	—	—		GF
				[M-H]^−^	345.1560	345.1555	1.45	165[M-H-C_6_H_10_O5-H_2_O]						
				[M+Cl]^−^	381.1327	381.1322	1.31	121[M−H−C_6_H_10_O_5_−CO_2_]						
13^* b*^	6.16	(+)-Catechin	C_15_H_14_O_6_	[M-H]^−^	289.0727	289.0718	3.11	245[M-H-CO_2_]	[M+H]^+^	291.0841	291.0863	-7.56		AF/RRR
				[M+Cl]^−^	325.0489	325.0484	1.54	247[M-H-C_2_H_2_O]						
								179[M-H-C_6_H_6_O_2_]						
								271[M-H-H_2_O]						
14	6.23	Gardenone	C_12_H_20_O_3_	[M+HCOO]^−^	257.1393	257.1394	-0.39	213[M-H-CO_2_]	—	—	—	—		GF
15 ^*b*^	6.30	Chlorogenic acid	C_16_H_18_O_9_	[M-H]^−^	353.0890	353.0878	3.40	191[M-H-C_9_H_6_O_3_]	[M+H]^+^	355.1003	355.1024	-5.91		GF
				[2M-H]^−^	707.1834	707.1829	0.71	179[M-H-C_9_H_10_O_5_]						
								135[M-H-C_8_H_10_O_7_]						
16	6.46	Darendoside A	C_19_H_28_O_11_	[M-H]^−^	431.1561	431.1559	0.46		—	—	—	—		SR
17 ^*b*^	6.74	Caffeic acid	C_9_H_8_O_4_	[M-H]^−^	179.0357	179.0350	3.91	135[M-H-CO_2_]	[M+H]^+^	181.0483	181.0495	-6.63		SR/EA
18	6.85	Cryptochlorogenic acid	C_16_H_18_O_9_	[M-H]^−^	353.0883	353.0878	1.42	179[M-H-C_9_H_10_O_5_]	—	—	—	—		GF
19	7.11	Genipin-1-*β*-gentiobioside	C_23_H_34_O_15_	[M-H]^−^	549.1830	549.1825	0.91	225[M-H-2C_6_H_10_O_5_]	[M+Na]^+^	573.1794	573.1790	0.70	541[M+Na-C_2_H_2_O]^+^	GF
				[M+HCOO]^−^	595.1879	595.1880	-0.17	207[M-H-2C_6_H_10_O_5_-H_2_O]	—	—	—	—		
20 ^*b*^	7.60	Syringin	C_17_H_24_O_9_	[M+HCOO]^−^	417.1401	417.1402	-0.24	373[M-H-CO_2_]	[M+Na]^+^	395.1300	395.1313	-3.29		RA
21 ^*b*^	8.11	Geniposide	C_17_H_24_O_10_	[M-H]^−^	387.1308	387.1297	2.84	225[M-H-C_6_H_10_O_5_]	[M+Na]^+^	411.1286	411.1262	5.84		GF
				[M+HCOO]^−^	433.1351	433.1352	-0.23	207[M-H-C_6_H_10_O_5_-H_2_O]						
								123[M-H-C_10_H_16_O_8_]						
								101[M-H-C_13_H_18_O_7_]						
22 ^*b*^	8.16	(-)-Epicatechin	C_15_H_14_O_6_	[M-H]^−^	289.0721	289.0718	1.04	245[M-H-CO_2_]	[M+H]^+^	291.0868	291.0863	1.72		AF
				[M+Cl]^−^	325.0488	325.0484	1.23	179[M-H-C_6_H_6_O_2_]	—	—	—	—		
23	8.17	Genipin	C_11_H_14_O_5_	[M-H]^−^	225.0772	225.0768	1.78	207[M-H-H_2_O]	[M+H]^+^	227.0917	227.0914	1.32	209[M+H-H_2_O]^+^	GF
								163[M-H-H_2_O-CO_2_]						
24	9.05	p-Coumaric acid	C_9_H_8_O_3_	[M-H]^−^	163.0406	163.0401	3.07	119[M-H-CO_2_]	[M+H]^+^	165.0547	165.0546	0.61	147[M+H-H_2_O]^+^	SR
25	9.06	Nicotiflorin	C_27_H_30_O_15_	[M-H]^−^	593.1518	593.1512	1.01	285[M-H-rha-glu]	[M+H]^+^	595.1657	595.1657	0.00		GF
								151[M-H-C_19_H_22_O_12_]						
26	9.22	Khelloside	C_19_H_20_O_10_	[M-H]^−^	407.1010	407.0984	6.39		[M+H]^+^	409.1119	409.1129	-2.44		AF
27	10.58	Schaftoside/Isoschaftoside	C_26_H_28_O_14_	[M-H]^−^	563.1407	563.1406	0.18	503[M-H-C_2_H_4_O_2_],	[M+H]^+^	565.1544	565.1552	-1.42	547[M+H-H_2_O]^+^	SR/EA
								443[M-H-2C_2_H_4_O_2_]					529[M+H-CO2]^+^	
28	11.25	Rhoifolin	C_27_H_30_O_14_	[M-H]^−^	577.1562	577.1563	-0.17		[M+H]^+^	579.1700	579.1708	-1.38		AF
29 ^*b*^	12.21	Rutin	C_27_H_30_O_16_	[M-H]^−^	609.1466	609.1461	0.82	301[M-H-rha-gla]	[M+H]^+^	611.1593	611.1607	-2.29	303[M+H-rha-glc]^+^	GF/EA
				—	—	—	—	151[M-H-C_20_H_27_O_12_]	[M+Na]^+^	633.1425	633.1426	-0.16		
								178[M-H-C_19_H_27_O_11_]						
30	12.60	Scutellarin	C_21_H_18_O_12_	[M-H]^−^	461.0724	461.0725	-0.22	285[M-H-glc]	[M+H]^+^	463.0877	463.0871	1.30		SR
31	12.74	Carthamidin	C_15_H_12_O_6_	—	—	—	—		[M+H]^+^	289.0693	289.0707	-4.84		SR
32	12.78	Neoeriocitrin	C_27_H_32_O_15_	[M-H]^−^	595.1671	595.1668	0.50		[M+H]^+^	597.1824	597.1814	1.67		AF
33	13.13	Isoquercitrin	C_21_H_20_O_12_	[M-H]^−^	463.0880	463.0882	-0.43	301[M-H-C_6_H_10_O_5_]	[M+H]^+^	465.1053	465.1028	5.38		GF/EA
								300[M-H-C_6_H_11_O_5_]						
34	13.66	Acteoside	C_29_H_36_O_15_	[M-H]^−^	623.1969	623.1981	-1.93	461[M-H-C_6_H_10_O_5_]	[M+Na]^+^	647.1916	647.1946	-4.64		SR
								315[M-H-rha]						
35	14.09	Narirutin	C_27_H_32_O_14_	[M-H]^−^	579.1709	579.1719	-1.73	271[M-H-rha-gla]	[M+H]^+^	581.1852	581.1865	-2.24		AF
				[M+Cl]^−^	615.1490	615.1486	0.65		[M+Na]^+^	603.1682	603.1684	-0.33		
36	14.36	Kaempferol	C_15_H_10_O_6_	[M-H]^−^	285.0410	285.0405	1.75	241[M-H-CO_2_]	[M+H]^+^	287.0544	287.0550	-2.09	153[M+H-C_8_H_6_O_2_]^+^	EA
37	14.86	Naringin	C_27_H_32_O_14_	[M-H]^−^	579.1728	579.1719	1.55	271[M-H-rha-gla]	[M+H]^+^	581.1847	581.1865	-3.10	435[M+H-C_6_H_10_O_4_]^+^	AF
				[M+Cl]^−^	615.1482	615.1486	-0.65	151[M-H-C_20_H_28_O_10_]	[M+Na]^+^	603.1672	603.1684	-1.99	273[M+H-rha-gla]^+^	
								119[M-H-C_19_H_24_O_13_]						
								107[M-H-C_20_H_28_O_10_-CO_2_]						
								259[M-H-rha-gla-C_3_O2]						
								203[M-H-rha-gla-C_3_O_2_-C_2_H_2_O]						
38	15.25	Isorhoifolin	C_27_H_30_O_14_	[M-H]^−^	577.1566	577.1563	0.52		[M+H]^+^	579.1701	579.1708	-1.21		AF
39	15.37	Jasminoside S/H/I	C_22_H_36_O_12_	[M+HCOO]^−^	537.2186	537.2189	-0.56	375[M-H-C_6_H_10_O_5_]	[M+Na]^+^	515.2085	515.2099	-2.72		GF
				[M+Cl]^−^	527.1899	527.1901	-0.38	167[M-H-2C_6_H_10_O_5_]	—	—	—	—		
				[M-H]^−^	491.2123	491.2134	-2.24		—	—	—	—		
40 ^*b*^	15.65	Hesperidin	C_28_H_34_O_15_	[M-H]^−^	609.1829	609.1825	0.66	301[M-H-gla-rha]	[M+H]^+^	611.1954	611.1970	-2.62	449[M+H-gla^ +^	AF
				[M+Cl]^−^	645.1600	645.1592	1.24		[M+Na]^+^	633.1781	633.1790	-1.42	303[M+H-gla-rha]^+^	
41 ^*b*^	16.44	Neohesperidin	C_28_H_34_O_15_	[M-H]^−^	609.1823	609.1825	-0.33	463[M-H-rha]	[M+H]^+^	611.1961	611.1970	-1.47	449[M+H-gla]^+^	AF
				[M+Cl]^−^	645.1587	645.1592	-0.78	301[M-H-gla-rha]	[M+Na]^+^	633.1782	633.1790	-1.26	303[M+H-gla-rha]^+^	
42	16.71	Viscidulin III	C_17_H_14_O_8_	[M-H]^−^	345.0626	345.0616	2.90	301[M-H-CO_2_]	[M+H]^+^	347.0748	347.0761	-3.75		SR
43 ^*b*^	17.60	Baicalin	C_21_H_18_O_11_	[M-H]^−^	445.0773	445.0776	-0.67	269[M-H-gluA]	[M+Na]^+^	469.0731	469.0741	-2.13		SR
				[2M-H]^−^	891.1628	891.1625	0.34	251[M-H-H_2_O]	[M+H]^+^	447.0920	447.0922	-0.45		
								241[M-H-CO]						
								225[M-H-CO_2_]						
								223[M-H-H_2_O-CO]						
								207[M-H-H_2_O-CO_2_]						
44	18.03	Crocin-1	C_44_H_64_O_24_	[M-H]^−^	975.3737	975.3715	2.26	651[M-H-2C_6_H_10_O_5_]	—	—	—	—		GF
				[M+Cl]^−^	1011.3496	1011.3482	1.38	327[M-H-4C6H10O5]	—	—	—	—		
45	18.694	Dihydrobaicalin	C_21_H_20_O_11_	[M-H]^−^	447.0942	447.0933	2.01	411[M-H-2H2O]	[M+H]^+^	449.1069	449.1078	-2.00		SR
				[2M-H]^−^	895.1931	895.2012	-9.05	271[M-H-glua]	[M+Na]^+^	471.0884	471.0898	-2.97		
								253[M-H-gluA-H_2_O]						
46	19.51	Cistanoside D	C_31_H_40_O_15_	[M-H]^−^	651.2281	651.2294	-2.00	475[M-H-gluA]	[M+Na]^+^	675.2240	675.2259	-2.81		SR
				[M+Cl]^−^	687.2063	687.2061	0.29		—	—	—	—		
47 ^*b*^	19.54	Quercetin	C_15_H_10_O_7_	[M-H]^−^	301.0363	301.0354	2.99	151[M-H-C_6_H_8_O_3_]	[M+H]^+^	303.0492	303.0499	-2.31	285[M+H-H_2_O]^+^	GF/EA
													257[M+H-H_2_O-CO]^+^	
48	20.13	Crocin-2	C_38_H_54_O_19_	[M-H]^−^	813.3186	813.3187	-0.12	651[M-H-C_6_H_10_O_5_]	[M+Na]^+^	837.3156	837.3152	0.48		GF
								489[M-H-2C_6_H_10_O_5_]						
								327[M-H-3C_6_H_10_O_5_]						
49	20.68	Wogonoside	C_22_H_20_O_11_	[M-H]^−^	459.0953	459.0933	4.36	283[M-H-gluA]	[M+H]^+^	461.1091	461.1078	2.82	443[M+H-H_2_O]^+^	SR
								268[M-H-gluA-CH_3_]	[M+Na]^+^	483.0879	483.0898	-3.93	285[M+H-gluA]^+^	
													270[M+H-gluA-CH3]^+^	
50	20.75	Cistanoside C	C_31_H_40_O_15_	[M-H]^−^	651.2295	651.2294	0.15	475[M-H-gluA]	[M+Na]^+^	675.2255	675.2259	-0.59		SR
				[M+Cl]^−^	687.2066	687.2061	0.73							
51	21.26	Pectolinarin	C_29_H_34_O_15_	[M-H]^−^	621.1812	621.1825	-2.09		[M+H]^+^	623.1958	623.1970	-1.93		AF
52	21.69	Baicalein O-gluA	C_22_H_20_O_11_	[M-H]^−^	459.0949	459.0933	3.49		[M+H]^+^	461.1086	461.1078	1.73		SR
		methylester		—	—	—	—		[M+Na]^+^	483.0886	483.0898	-2.48		
53	23.62	Hesperetin	C_16_H_14_O_6_	[M-H]^−^	301.0723	301.0718	1.66		[M+H]^+^	303.0851	303.0863	-3.96		AF
54	23.99	Tenaxin II	C_16_H_12_O_6_	[M-H]^−^	299.0569	299.0561	2.68	284[M-H-CH_3_]	[M+H]^+^	301.0701	301.0707	1.99	-286[M+H-CH_3_]^+^	SR
55 ^*b*^	24.20	Baicalein	C_15_H_10_O_5_	[M-H]^−^	269.0455	269.0455	0.00	251[M-H-H_2_O]	[M+H]^+^	271.0579	271.0601	-8.12		GF
								241[M-H-CO]						
								181[M-H-CO-O-CO_2_]						
								225[M-H-CO-O]						
								223[M-H-H_2_O-CO]						
56	24.49	Crocin-4	C_44_H_64_O_24_	[M-H]^−^	975.3725	975.3715	1.03		—	—	—	—		GF
57	24.89	Tenaxin II isomer	C_16_H_12_O_6_	[M-H]^−^	299.0558	299.0561	-1.00	284[M-H-CH_3_]	[M+H]+	301.0697	301.0707	-3.32	286[M+H-CH_3_]^+^	SR
58	25.72	Meranzin	C_15_H_16_O_4_	—	—	—	—		[M+Na]^+^	283.0940	283.0941	-0.35		AF
59	26.03	Dikamaliartanes A	C_30_H_44_O_6_	—	—	—	—		[M+Na]^+^	523.3043	523.3030	2.48	239[M+H-CO_2_]^+^	GF
60 ^*b*^	26.02	Aloe-emodin	C_15_H_10_O_5_	[M-H]^−^	269.0466	269.0455	4.09	239[M-H-CH_2_O]	[M+H]+	271.0589	271.0601	-4.43		RRR
								211[M-H-CO]						
								183[M-H-CO-CO]						
61 ^*b*^	26.71	Rhein	C_15_H_8_O_6_	[M-H]^−^	283.0256	283.0248	2.83	255[M-H-CO]	—	—	—	—		RRR
								239[M-H-CO_2_]						
								183[M-H-CO_2_-2CO]						
								211[M-H-CO_2_-CO]						
								183[M-H-CO-2CO]						
62	26.76	Limonin	C_26_H_30_O_8_	[M-H]^−^	469.1875	469.1868	1.49		[M+H]^+^	471.2006	471.2013	-1.49		AF
				[M+Cl]^−^	505.1631	505.1635	-0.79		—	—	—	—		
63	27.11	Skullcapflavone	C_18_H_16_O_7_	[M-H]^−^	343.0825	343.0823	0.58		[M+H]^+^	345.0960	345.0969	-2.61		SR
64 ^*b*^	27.20	Wogonin	C_16_H_12_O_5_	[M-H]^−^	283.0620	283.0612	2.83	240[M-H-CH_3_-COH]	[M+H]^+^	285.0745	285.0757	-4.21		SR
								239[M-H-CH_3_-COH]						
								223[M-H-CH_3_-CO_2_H]						
								212[M-H-CH_3_-2CO]						
65	27.86	Skullcapflavon II	C_19_H_18_O_8_	[M-H]^−^	373.0938	373.0929	2.41	358[M-H-CH_3_]	[M+H]^+^	375.1073	375.1074	-0.27	345[M+H-2CH_3_]^+^	SR
								343[M-H-2CH_3_]						
								257[M-H-4CH_3_-2CO]						
								328[M-H-3CH_3_]						
								300[M-H-3CH_3_-CO]						
								272[M-H-3CH_3_-2CO]						
66	27.87	Oroxylin A	C_16_H_12_O_5_	[M-H]^−^	283.0619	283.0612	2.47	268[M-H-CH_3_]	[M+H]^+^	285.0754	285.0757	-1.05		SR
								239[M-H-COH]						
67	28.67	Tenaxin I	C_18_H_16_O_7_	[M-H]^−^	343.0831	343.0823	2.33	328[M-H-CH_3_]	[M+H]^+^	345.0963	345.0969	-1.74		SR
								313[M-H-2CH_3_]						
								298[M-H-3CH_3_]						
68 ^*b*^	30.24	Emodin	C_15_H_10_O_5_	[M-H]^−^	269.0453	269.0455	-0.74	251[M-H-H_2_O]	[M+H]^+^	271.0600	271.0601	-0.37		RRR/EA
								241[M-H-CO]						
								225[M-H-CO-O]						
								181[M-H-CO-O-CO_2_]						
69	31.27	Costunolide	C_15_H_20_O_2_	—	—	—	—		[M+H]^+^	233.1535	233.1536	-0.43	187[M+H-CH_2_O_2_]^+^	RA
													215[M+H-H_2_O]^+^	
													159[M+H-C_3_H_6_O_2_]^+^	
70 ^*b*^	31.80	Dehydrocostuslactone	C_15_H_18_O_2_	—	—	—	—		[M+H]^+^	231.1357	231.1380	-9.95	185[M+H-CH_2_O_2_^+^	RA
													213[M+H-H_2_O]^+^	
													157[M+H-C_3_H_6_O_2_]^+^	
													195[M+H-H_2_O_4_]^+^	
													175[M+H-C_4_H_8_]^+^	
71 ^*b*^	32.99	Chrysophanol	C_15_H_10_O_4_	—	—	—	—		[M+H]^+^	255.0638	255.0652	-5.49		RRR
														
														
72 ^*b*^	34.51	Physcion	C_16_H_12_O_5_	—	—	—	—		[M+H]^+^	285.0765	285.0757	2.81		RRR
														

^a^ RRR, Rhei Radix et Rhizoma; EA, Lysimachiae Herba; SR, Scutellariae Radix; GF, Gardeniae Fructus; AR, Aucklandiae Radix; AF, Aurantii Fructus.

^b^ Components identified with reference compounds comparison.

## Data Availability

The data used to support the findings of this study are available from the corresponding author upon request.

## Supplementary Materials

Supplementary Materials Table S1: therapeutic targets of cholelithiasis; Table S2: compound targets for QRLDD; Table S3: pathway enrichment analysis of QRLDD-Disease targets network.
